# Antioxidant and Mitochondria-Targeted Activity of Caffeoylquinic-Acid-Rich Fractions of Wormwood (*Artemisia absinthium* L.) and Silver Wormwood (*Artemisia ludoviciana* Nutt.)

**DOI:** 10.3390/antiox10091405

**Published:** 2021-09-01

**Authors:** Justina Kamarauskaite, Rasa Baniene, Lina Raudone, Gabriele Vilkickyte, Rimanta Vainoriene, Vida Motiekaityte, Sonata Trumbeckaite

**Affiliations:** 1 Department of Pharmacognosy, Medical Academy, Lithuanian University of Health Sciences, Sukileliu Av. 13, LT-50162 Kaunas, Lithuania; Lina.Raudone@lsmuni.lt (L.R.); Sonata.Trumbeckaite@lsmuni.lt (S.T.); 2 Laboratory of Biopharmaceutical Research, Institute of Pharmaceutical Technologies, Lithuanian University of Health Sciences, Sukileliu Av. 13, LT-50162 Kaunas, Lithuania; gabriele.vilkickyte@lsmu.lt; 3 Neuroscience Institute, Lithuanian University of Health Sciences, Sukileliu Av. 13, LT-50162 Kaunas, Lithuania; Rasa.Baniene@lsmuni.lt; 4 Department of Biochemistry, Medical Academy, Lithuanian University of Health Sciences, Eiveniu Str. 4, LT-50161 Kaunas, Lithuania; 5 Institute of Regional Development, Vilnius University Siauliai Academy, Vytauto Str. 84, LT-76352 Siauliai, Lithuania; rimanta.vainoriene@su.lt; 6 Faculty of Public Governance and Business, Mykolas Romeris University, Ateities Str. 20, LT-08303 Vilnius, Lithuania; vmotiek@gmail.com

**Keywords:** caffeoylquinic acids, antioxidant effect, fractionation, mitochondria, cytochrome c, *Artemisia* spp.

## Abstract

Caffeoylquinic acids are some of the chemophenetically significant specialized metabolites found in plants of the family *Asteraceae* Dumort., possessing a broad spectrum of biological activities. As they might be potential mitochondria-targeted antioxidants, effective preparation methods—including extraction, isolation, and purification of caffeoylquinic acids from plant sources—are in great demand. The aim of this study was to fractionate the caffeoylquinic acids from cultivated wormwood (*Artemisia absinthium* L.) and silver wormwood (*Artemisia ludoviciana* Nutt.) herb acetone extracts and evaluate their phytochemical profiles, antioxidant activity (radical scavenging and reducing activities), effects on kidney mitochondrial functions, and cytochrome-c-reducing properties. The main findings of our study are as follows: (1) Aqueous fractions purified from wormwood and silver wormwood herb acetone extracts are rich in monocaffeoylquinic acids (chlorogenic acid, neochlorogenic acid, 4-*O*-caffeoylquinic acid), while methanolic fractions purified from wormwood and silver wormwood herb acetone extracts are rich in dicaffeoylquinic acids (4,5-dicaffeoylquinic acid, 3,4-dicaffeoylquinic acid, 3,5-dicaffeoylquinic acid). Aqueous fractions purified from wormwood and silver wormwood herb acetone extracts were solely composed of monocaffeoylquinic acids. Methanolic fractions purified from wormwood and silver wormwood herb acetone extracts contained only dicaffeoylquinic acids. (2) Fractions purified from silver wormwood herb acetone extracts stood out as having the greatest content of caffeoylquinic acids. (3) The greatest radical scavenging activity was determined in the dicaffeoylquinic-acid-rich fraction purified from silver wormwood herb acetone extract; the greatest reducing activity was determined in the dicaffeoylquinic-acid-rich fraction purified from wormwood herb acetone extract. (4) The effect of both fractions on mitochondrial functions was dose-dependent; lower concentrations of caffeoylquinic-acid-rich fractions had no effect on mitochondrial functions, whereas higher concentrations of caffeoylquinic-acid-rich fractions reduced the state 3 respiration rate (with the complex-I-dependent substrate glutamate/malate). (5) Both monocaffeoylquinic- and dicaffeoylquinic-acid-rich fractions possessed cytochrome-c-reducing properties; the greatest cytochrome c reduction properties were determined in the dicaffeoylquinic-acid-rich fraction purified from wormwood herb acetone extract. In summary, these findings show that caffeoylquinic acids might be beneficial as promising antioxidant and cytochrome-c-reducing agents for the modulation of mitochondria and treatment of various mitochondrial-pathway-associated pathologies.

## 1. Introduction

*Artemisia* L. (*Asteraceae* Dumort.) is a tribe of small herbs and shrubs distributed in the northern temperate areas [1]. Various species have aroused great interest in pharmaceutical and food production, generating much research on the most varied aspects of *Artemisia* plants [2]. *Artemisia* species possess antibacterial, antiviral [3], antiparasitic, antifungal [4], nematicidal, and insecticidal properties, which generally contribute significantly to the safeguarding of the plant [5,6]. These biological effects are due to the extremely rich phytochemical composition of the genus *Artemisia*, which contains a body of phytochemical compounds of different chemical origins—namely, essential oil components (e.g., γ-terpinene, 1,4-terpeniol, trans-thujone, etc.), organic acids, resins, tannins, and a great profile of phenolic compounds [7]. Caffeoylquinic acids formed as ester molecules by the combination of caffeic acid and (−)-quinic acid [8] are characteristic compounds of the *Asteraceae* family [9,10]; they possess antioxidant, antibacterial, anticancer, antihistaminic, and other biological effects [11,12]. Due to their wide range of biological effects, the study of caffeoylquinic acids’ phytochemical profiles and the validation of their biological effects are relevant, innovative, and interesting in the scientific field. Therefore, effective procedures of preparation for caffeoylquinic acids—including their extraction, isolation and purification from plant sources—are in great demand. 

Today, increasing interest is shown towards mitochondria-targeted antioxidants, since they can serve as potentially effective therapeutic agents for various pathologies. Mitochondria are important organelles that generate and supply most of the cell’s energy via oxidative phosphorylation that takes place in the inner membrane, and they also regulate apoptotic pathways; therefore, the regulation of their function by biologically active compounds may have important therapeutic significance [13].

In the cell, mitochondria are a relevant source of reactive oxygen species (ROS) [14]. ROS are important second messengers that regulate signal transduction pathways and are critical to cell proliferation and death [15]. It is well known that increased cellular O_2_ concentration or decreased activity of antioxidant enzymes causes oxidative stress. Excessive ROS have damaging effects on cellular components, such as DNA and proteins, and can cause lipid oxidation [16] as well as other cytotoxic effects, leading to aging and many diseases, including cancer, cardiovascular and neurodegenerative diseases, and diabetes [17,18].

One of the most important sources of antioxidants is plant polyphenols, which can reduce the occurrence of oxidative stress, acting primarily as ROS scavengers or modulators of ROS-removing enzymes [19,20,21,22,23]. Phenolic compounds such as caffeoylquinic acids might be potential mitochondria-targeted antioxidants; however, their effects on mitochondrial function have not yet been studied. Bioactive compounds that might regulate redox state in the cell are also of great importance, as their ability to reduce a component of the electron transport chain (cytochrome c) in the mitochondria may prevent apoptotic cell death [24,25].

The aim of this study was to fractionate caffeoylquinic acids from cultivated wormwood (*A. absinthium*) and silver wormwood (*A. ludoviciana*) herb acetone extracts and evaluate their qualitative and quantitative profiles in fractions, as well as their antioxidant activity, effect on kidney mitochondrial functions, and cytochrome-c-reducing properties. To the best of our knowledge, there are no previous comparative data on the presence of caffeoylquinic acids in wormwood (*A. absinthium*) and silver wormwood (*A. ludoviciana*) and their possible relationship with the antioxidant and mitochondrial activity of fractions obtained from these plants’ herb acetone extracts.

## 2. Materials and Methods

### 2.1. Chemicals and Solvents

HPLC standard compounds—chlorogenic acid, neochlorogenic acid, 4-*O*-caffeoylquinic acid, 4,5-dicaffeoylquinic acid, 3,4-dicaffeoylquinic acid, 3,5-dicaffeoylquinic acid, luteolin-7-glucoside, and luteolin-7-rutinoside—were purchased from Sigma-Aldrich (Steinheim, Germany). 

Chemicals for antioxidant activity, mitochondrial respiration, and cytochrome-c-reducing activity—Trolox (6-hydroxy-2,5,7,8-tetramethylchroman-2-carboxylic acid), ABTS (2,2′-azino-bis(3-ethylbenzothiazoline-6-sulfonic acid) diammonium salt), sodium acetate (CH_3_COONa), TPTZ (2,4,6-tri-(2-pyridyl)-S-triazine), ferric chloride (FeCl_3_), sucrose, TrisHCl (Trizma hydrochloride), EDTA (ethylenediaminetetraacetic acid), KCl (potassium chloride), KH_2_PO_4_ (Potassium phosphate monobasic), MgCl_2_ × 6H_2_O (Magnesium chloride hexahydrate), L-(-)-Malic acid, ADP (adenosine 5′-diphosphate sodium salt), cytochrome c from equine heart, and dithionite (sodium hydrosulfite)—were purchased from Sigma-Aldrich (Steinheim, Germany). K_2_S_2_O_8_ (potassium persulfate) was purchased from Alfa Aesar (Karlsruhe, Germany). L-Glutamic acid was purchased from Fluka Analytical (Munich, Germany). All standard substances and chemicals were of analytical grade.

The substances 99.9% acetonitrile, 99.9% acetone, 99.9% methanol, 99.8% anhydrous acetic acid, and 37% hydrochloric acid (HCl) were obtained from Sigma-Aldrich (Steinheim, Germany), 99.8% trifluoroacetic acid was obtained from Merck (Darmstadt, Germany), and 96% ethanol was obtained from Vilniaus Degtine SC (Vilnius, Lithuania). The water was purified using a Millipore Milli-Q apparatus.

### 2.2. Plant Material

In Lithuania, wormwood (*Artemisia absinthium* L.) is present in all districts, but is more prevalent in the southern and eastern parts of the country. Sometimes it is cultivated as a medicinal plant. Silver wormwood (*Artemisia ludoviciana* Nutt.) is cultivated as an ornamental plant in Lithuania. Both *Artemisia* species analyzed in the present study (i.e., wormwood (*A. absinthium*) and silver wormwood (*A. ludoviciana*)) were cultivated and collected during the flowering season at Siauliai Academy of Vilnius University Botanical Garden, Siauliai, Republic of Lithuania. The geographical coordinates of the botanical garden are 55°55′57″ N, 23°16′59″ E (WGS). The analyzed wormwood (*A. absinthium*) specimens originated from a wild habitat in Prienai District, Lithuania; this species has been cultivated in the botanical garden since 2002. The propagation material of analyzed silver wormwood (*A. ludoviciana*) specimens was received from Vytautas Magnus University Botanical Garden, Kaunas, Republic of Lithuania; this species has been cultivated in the botanical garden since 2000. The flowering seasons of the analyzed species differ markedly. In 2019, raw wormwood (*A. absinthium)* was collected on 25 July, while raw silver wormwood (*A. ludoviciana*) was collected on 10 September. In 2020, the flowering season of silver wormwood (*A. ludoviciana*) started at a similar time (7 July). A very long period (~2 months) between the budding and flowering seasons of silver wormwood (*A. ludoviciana*) was observed (budding season starts in the beginning of July). The plant cuttings were air-dried at room temperature, and subsequently stored in paper sacks until analysis.

### 2.3. Production of Dry Extracts

The raw material was ground and used for extraction. The production of dry extracts was performed as described by Vilkickyte et al. [26], with some modifications. A precise weight of 30 g of shredded plant matter was extracted with 250 mL of 70% aqueous acetone via ultrasonication for 15 min, at a frequency 80 kHz, power of 30 Watts, and 27 °C temperature. The suspension was filtered through a vacuum filter. The extraction procedure was repeated four times, and the suspensions were combined after each filtration. Filtrates were evaporated at 40 °C until the acetone was removed, and then lyophilization was applied to obtain dry extracts of *Artemisia* plants.

### 2.4. Fractionation of Caffeoylquinic Acids

Fractionation of caffeoylquinic acids was performed as described by Vilkickyte et al. [26], with some modifications. Briefly, ~5 g (precise weight) of dry extracts of *Artemisia* herb were dissolved in 200 mL of 50% methanol and applied to a glass column packed with Sephadex LH-20. Distilled water (500 mL; 250 mL two times) was used to fractionate the monocaffeoylquinic acids, while 70% aqueous methanol (1000 mL; 250 mL four times) was used to fractionate the dicaffeoylquinic acids. The obtained fractions were evaporated in an IKA RV 10 rotary evaporator and lyophilized in a Telstar LyoQuest freeze dryer to obtain dry fractions.

### 2.5. HPLC-PDA Analysis for Characterization of Caffeoylquinic Acids and GC–MS Analysis for Essential Oil Components

The phenolic profiles of the fractions were determined using HPLC (Waters e2695 Alliance system, Waters, Milford, MA, USA) coupled with a PDA detector. The separations were performed as described by Vilkickyte et al. [26], with minor modifications. Briefly, an ACE Super C18 (250 mm × 4.6 mm, 3 µm) column (ACT, Aberdeen, UK) was used, and the flow rate was 0.5 mL/min. The column temperature was set to 15 °C, with an injection volume of 10 µL. The mobile phase consisted of 0.1% trifluoroacetic acid (A) and acetonitrile (B), and the gradient solution pattern was 0 min, 85% A; 0–30 min, 70% A; 30–50 min, 40% A; 50–56 min, 10% A; 56–65 min, 15% A. To prepare the HPLC standard curves, each test compound was weighed out accurately, dissolved in 70% methanol (*v*/*v*) at a concentration of 1 mg/mL, and filtered through a 0.2 µm pore size PVDF syringe filter (Macherey-Nagel GmbH & Co. KG, Düren, Germany). Chromatographic peak identification was performed by comparing the analyte and reference compound (individual standard reference compounds of caffeoylquinic acids) retention time and the UV absorption spectra (Table A1). Quantification was performed at 320 nm for caffeoylquinic acids and at 350 nm for luteolin glycosides. For quantification, 5–7-point calibration curves were constructed by plotting the response of each analyte versus concentration (Table A1). 

The gas chromatography–mass spectrometry (GC-MS) research was performed using a headspace methodology on a SHIMADZU GC-MS-QP2010 Ultra chromatography system with an Rxi-5ms (Restek Corporation, Benner Circle, Bellefonte, PA, USA) fused silica capillary column (30 m × 0.25 mm, 0.25 µm film coating). The oven temperature was programmed at 40 °C for 2 min, then increased by 3 °C /min to 150 °C, then 2 °C/min to 185 °C, then 10 °C/min to 250 °C, and then 20 °C/min to 310 °C for 5 min. The injector temperature was 260 °C, in split injection mode, with an injection volume of 1 µL and a split ratio of 1:5. The mass spectra scan range of m/z was 29–500 amu, with a mass scan time of 0.2 s, interface temperature of 280 °C, and IonSource temperature of 200 °C. Identification of the active compounds was done on the basis of retention index and library mass search database (NIST14, NIST14s, WR10, WR10R).

### 2.6. Antioxidant Activity of Caffeoylquinic-Acid-Rich Fractions and Acetone Extracts

For antioxidant activity evaluation, all tested fractions and acetone extracts were dissolved in 70% ethanol until complete dissolution, obtaining a concentration of 0.5 mg/mL. Spectrophotometric assays were implemented as described by Raudone et al. [27] and in our previous report [28]. 

Briefly, for the ABTS assay, 3 mL of diluted ABTS working solution was mixed with test solutions (10 µL), and absorbance was measured at 734 nm using a spectrophotometer. The results were expressed as µM TE/g DW (micromolar of standard antioxidant Trolox equivalents per gram of dry weight of fractions). Trolox calibration curve y = 0.0001x + 0.0248; determination coefficient R^2^ = 0.9992.

Briefly, for the FRAP assay, 3 mL freshly prepared FRAP reagent was mixed with test solutions (10 µL), and absorbance was recorded at 593 nm. The results were expressed in the same way as in the ABTS assay. Trolox calibration curve y = 0.0001x + 0.0046; determination coefficient R^2^ = 0.9988.

### 2.7. Preparation of Isolated Kidney Mitochondria

Preparation of isolated kidney mitochondria of *Wistar* rats was performed using a differential centrifugation method, as described in [28].

### 2.8. Measurement of Mitochondrial Respiration

The mitochondrial functions were measured using an Oxygraph-2k high-resolution respirometry system (OROBOROS Instruments, Innsbruck, Austria) in the incubation medium, as described in [28]. The routine mitochondrial respiration (V_0_) was recorded in the medium supplemented with mitochondria and 5 mM glutamate + 5 mM malate as respiratory substrates. The state 3 respiration rate (V_ADP_) was determined following the addition of 1 mM ADP. The exogenous cytochrome c (32 µM) was added to the incubation vessel during the state 3 respiration to determine the intactness of the mitochondrial outer membrane. The respiratory control index (RCI) for glutamate/malate was calculated as the ratio between the V_ADP_ and V_0_ respiration rates. Cytochrome c’s effect was calculated as the ratio between V_ADP+cyt c_ and V_ADP_. Mitochondrial respiration rates were expressed as pM O/s/0.25 mg of protein.

All stock solutions of fractions that were used in our study for the investigation of mitochondrial function were prepared in water—specifically, the lyophilized extract obtained from the prepared fractions was dissolved in water. The direct impact of caffeoylquinic-acid-rich fractions on mitochondrial functionality was analyzed in isolated kidney mitochondria incubated with two different concentrations (0.008 µg/mL and 0.8 µg/mL) of caffeoylquinic-acid-rich fractions. Specifically, the 1 µL fraction aqueous solution was added to the 2 mL incubation vessel before adding the mitochondrial suspension. The control group represents data without added aqueous solution of fractions. Table 1 presents the end concentration of caffeoylquinic acids in 1 mL of incubation vessel after addition of 1 µL of each fraction’s aqueous solution. The end concentration (ng/mL) of each compound was calculated using the formula:

$$X=\frac{A*B}{10^{3}}$$

where *X* is the concentration of caffeoylquinic acid (ng/mL); *A* is the identified amount (µg) of caffeoylquinic acid in 1 g of lyophilized fraction; and *B* is the amount (µg) of lyophilized fraction in 1 mL.

### 2.9. Measurement of Cytochrome c Reduction Level

The reduction of cytochrome c was recorded spectrophotometrically using a Helios Gama spectrophotometer as described in our previous study [28]. Briefly, the absorption spectra at 500–600 nm of various fractions in 1 mL of incubation medium were measured, and cytochrome c (20 µM) was added to the same buffer. After that, caffeoylquinic acid fractions (32.5 µg/mL and 65 µg/mL) were added and registered every 3 min for 21 min.

All stock solutions of fractions and acetone extracts that were used in our study were prepared in water—specifically, the lyophilized extracts obtained from the prepared fractions or acetone extracts were dissolved in water. Two different concentrations (32.5 µg/mL or 65 µg/mL) of caffeoylquinic-acid-rich fractions were used to evaluate the cytochrome c reduction properties. Table 2 presents the end concentrations of caffeoylquinic acids in 1 mL of incubation cuvette after the addition of 5 µL of each fraction’s or acetone extracts aqueous solution. The end concentration (ng/mL) of each compound was calculated by the same formula mentioned in Section 2.8.

### 2.10. Statistical Analysis 

Data were analyzed using SPSS 25.0 software by applying both one-way and two-way ANOVA (data on the effects of fractions on cytochrome c reduction), followed by Fisher’s LSD post hoc test, and presented as the mean ± SD (±SEM in biological experiments) of 3–5 individual experiments. The means of individual experiments were obtained from at least three repeated measurements. Student’s *t*-test was applied to compare the differences between phytochemical analysis data of caffeoylquinic-acid-rich fractions. *p* < 0.05 was considered statistically significant.

## 3. Results

### 3.1. Phytochemical Analysis of Caffeoylquinic-Acid-Rich Fractions and Acetone Extracts

**Figure 1:**
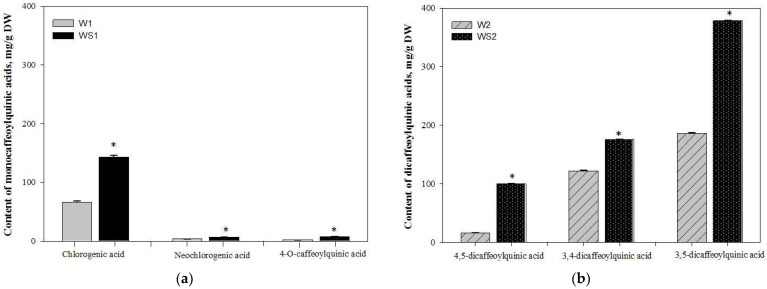
(**a**) Content of monocaffeoylquinic acids (mg/g) in the aqueous fractions, marked W1 and WS1, from wormwood and silver wormwood herb acetone extracts, respectively, and (**b**) content of dicaffeoylquinic acids in the methanolic fractions, marked W2 and WS2, from wormwood and silver wormwood herb acetone extracts, respectively. * *p* < 0.05 vs. the caffeoylquinic acid composition of fractions W1 and W2. Fraction W1 is the aqueous monocaffeoylquinic-acid-rich fraction from wormwood herb acetone extract; fraction WS1 is the aqueous monocaffeoylquinic-acid-rich fraction from silver wormwood herb acetone extract; fraction W2 is the methanolic dicaffeoylquinic-acid-rich fraction from wormwood herb acetone extract; fraction WS2 is the methanolic dicaffeoylquinic-acid-rich fraction from silver wormwood herb acetone extract.

**Figure 2:**
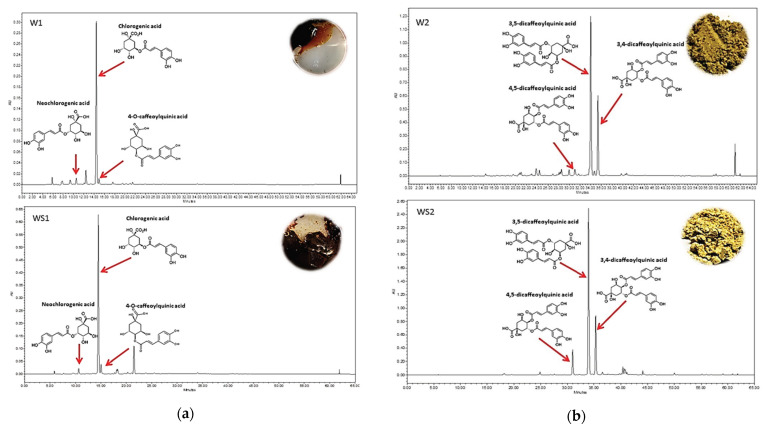
HPLC-PDA profiles of caffeoylquinic acids in the (**a**) monocaffeoylquinic-acid-rich fractions (aqueous fractions) from wormwood and silver wormwood herb acetone extracts—marked W1 and WS1, respectively—and in the (**b**) dicaffeoylquinic-acid-rich fractions (methanolic fractions) from wormwood and silver wormwood herb acetone extracts—marked W2 and WS2, rspectively—detected at 320 nm. Fraction W1 is the aqueous monocaffeoylquinic-acid-rich fraction from wormwood herb acetone extract; fraction WS1 is the aqueous monocaffeoylquinic-acid-rich fraction from silver wormwood herb acetone extract; fraction W2 is the methanolic dicaffeoylquinic-acid-rich fraction from wormwood herb acetone extract; fraction WS2 is the methanolic dicaffeoylquinic-acid-rich fraction from silver wormwood herb acetone extract.

Eight components have been identified in wormwood herb total acetone extract, and six components in silver wormwood herb total acetone extract. Chromatograms and determined amounts of the identified compounds are given in Figures S1 and S2. Later, four fractions were obtained from herb acetone extracts during the experiments, and six major antioxidant components of wormwood and silver wormwood were isolated and identified as caffeoylquinic acids. Their qualitative profiles differed significantly (*p* < 0.05). Fractionation increased levels of caffeoylquinic acids by concentrating them during washing steps, as compared with total acetone extracts from wormwood and silver wormwood (Table S1). Aqueous fractions from wormwood and silver wormwood herb acetone extracts—marked W1 and WS1, respectively—were characterized only with monocaffeoylquinic acids (chlorogenic acid, neochlorogenic acid, 4-*O*-caffeoylquinic acid). Methanolic fractions from wormwood and silver wormwood herb acetone extracts—marked W2 and WS2, respectively—were characterized only with dicaffeoylquinic acids (4,5-dicaffeoylquinic acid, 3,4-dicaffeoylquinic acid, 3,5-dicaffeoylquinic acid). A comparison of the caffeoylquinic acids’ profiles in the fractions is shown in Figure 1 and Figure 2. The aqueous monocaffeoylquinic-acid-rich fraction from wormwood herb acetone extract (marked W1) contained the lowest amounts of these compounds (66.8 ± 1.7 mg/g DW chlorogenic acid, making up 91.8% of the total identified monocaffeoylquinic acids; 3.8 ± 0.1 mg/g DW neochlorogenic acid, making up 5.2% of the total identified monocaffeoylquinic acids; and 2.2 ± 0.1 mg/g DW 4-*O*-caffeoylquinic acid, making up 3.0% of the total identified monocaffeoylquinic acids) (Figure 1a), *p* < 0.05. The highest amounts of monocaffeoylquinic acids were found in the aqueous monocaffeoylquinic-acid-rich fraction from silver wormwood herb acetone extract (marked WS1), which contained 143.3 ± 2.8 mg/g DW chlorogenic acid, making up 90.1% of the total identified monocaffeoylquinic acids; 6.7 ± 0.1 mg/g DW neochlorogenic acid, making up 4.2% of the total identified monocaffeoylquinic acids; and 7.8 ± 0.2 mg/g DW 4-*O*-caffeoylquinic acid, making up 4.9% of the total identified monocaffeoylquinic acids (Figure 1a), *p* < 0.05. The highest amounts of caffeoylquinic acids were found in methanolic dicaffeoylquinic-acid-rich fractions from wormwood and silver wormwood herb acetone extracts (marked W2 and WS2, respectively). The methanolic dicaffeoylquinic-acid-rich fraction (marked W2) contained less dicaffeoylquinic acids (16.3 ± 0.6 mg/g DW 4,5-dicaffeoylquinic acid, making up 5.0% of the total identified dicaffeoylquinic acids; 122.3 ± 0.7 mg/g DW 3,4-dicaffeoylquinic acid, making up 37.6% of the total identified dicaffeoylquinic acids; and 186.5 ± 0.9 mg/g DW 3,5-dicaffeoylquinic acid, making up 57.4% of the total identified dicaffeoylquinic acids) (Figure 1b), *p* < 0.05. The highest amounts of dicaffeoylquinic acids were found in the fraction from silver wormwood herb acetone extract (marked WS2) (101.1 ± 0.6 mg/g DW 4,5-dicaffeoylquinic acid, making up 15.4% of the total identified dicaffeoylquinic acids; 175.9 ± 0.5 mg/g DW 3,4-dicaffeoylquinic acid, making up 26.8% of the total identified dicaffeoylquinic acids; and 378.8 ± 0.5 mg/g DW 3,5-dicaffeoylquinic acid, making up 57.8% of the total identified dicaffeoylquinic acids) (Figure 1b), *p* < 0.05.

No other active compounds were detected in the fractions via GC-MS (Figure S3). This indicates that the preparation of the fractions ensures the absence of essential oil components. We thus confirm the hypothesis that, due to the many technological stages throughout the production of the fractions, essential oil components were removed.

### 3.2. Antioxidant Activity of Fractions and Acetone Extracts

The radical scavenging and reducing capacities of the caffeoylquinic-acid-rich fractions when applying the ABTS and FRAP assays are reported in Table 3 by their TE/g DW values. The highest radical scavenging activity was observed in the methanolic dicaffeoylquinic-acid-rich fraction from silver wormwood herb acetone extract (marked WS2, 1693 ± 59 μM TE/g DW, *p* < 0.05 as compared with the fractions marked W1, WS1, and W2. The radical scavenging activity of the aqueous monocaffeoylquinic-acid-rich fraction from silver wormwood herb acetone extract (marked WS1) was 745 ± 83 μM TE/g DW, *p* < 0.05 as compared with the fractions marked W1 and WS2. A similar antiradical activity was observed in the methanolic dicaffeoylquinic-acid-rich fraction from wormwood herb acetone extract (marked W2) (679 ± 134 μM TE/g DW), *p* < 0.05 as compared with the fractions marked W1 and WS2. The lowest radical scavenging activity was observed in the W1 fraction (367 ± 58 μM TE/g DW), *p* < 0.05 as compared with the fractions marked WS1, W2, and WS2 (Table 3). 

In the FRAP assay, the highest reducing activity was determined in the methanolic dicaffeoylquinic-acid-rich fraction from wormwood herb acetone extract (marked W2) (6952 ± 162 μM TE/g DW), *p* < 0.05 as compared with the fractions marked W1, WS1, and WS2. However, the evaluation of the reducing activity of the examined caffeoylquinic-acid-rich fractions in vitro revealed that the reducing activity in the W1, WS1, and WS2 fractions was 5385 ± 168 μM TE/g DW (*p* < 0.05 as compared with the fractions marked W2 and WS2), 5505 ± 175 μM TE/g DW (*p* < 0.05 as compared with the fractions marked W2 and WS2), and 6052 ± 81 μM TE/g DW (*p* < 0.05 as compared with the fractions marked W1, WS, and W2), respectively (Table 3).

We also evaluated the radical scavenging and reducing activities of wormwood and silver wormwood herb acetone extracts. The radical scavenging activity was 795 ± 26 µM TE/g DW in acetone extract from silver wormwood herb and 317 ± 26 µM TE/g DW in acetone extract from wormwood herb. In the FRAP assay, the reducing activity was lower in both acetone extracts from silver wormwood (1117 ± 85 µM TE/g DW) and wormwood (214 ± 24 µM TE/g DW) herb as compared with the caffeoylquinic-acid-rich fractions purified from these plants’ herb acetone extracts (*p* < 0.05).

### 3.3. Effects of Fractions on Kidney Mitochondrial Respiration Rates

**Figure 3:**
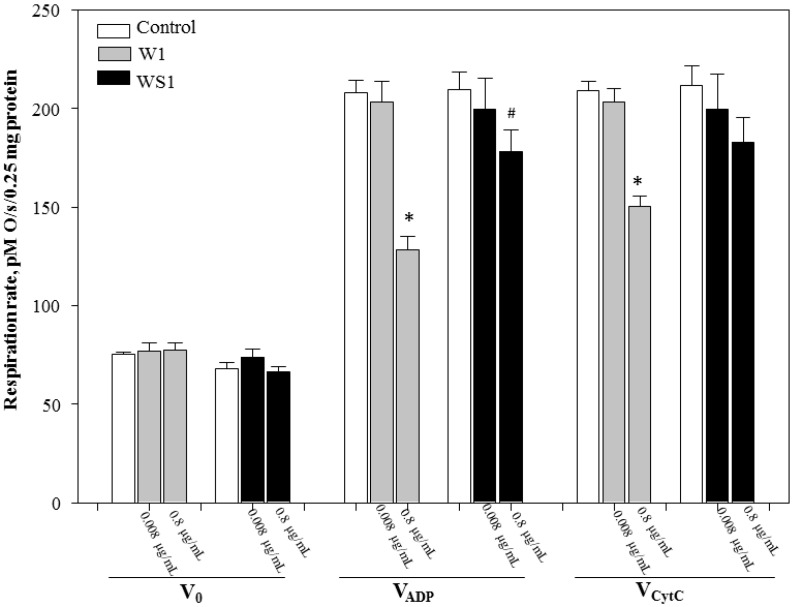
Effect of aqueous monocaffeoylquinic-acid-rich fractions from wormwood and silver wormwood herb acetone extracts on mitochondrial routine respiration rate, V_0_ (state 2 respiration rate in the presence of 0.25 mg/mL mitochondria and the substrates 5 mM glutamate + 5 mM malate), V_ADP_, (state 3 respiration rate in the presence of the substrates 5 mM glutamate + 5 mM malate and 1 mM ADP) and V_cyt C_, (state 3 respiration rate in the presence of 32 µM cytochrome c). *: *p* < 0.05 vs. respective control; #: *p* < 0.05 vs. fraction from wormwood (marked W1) at the same concentration. In the W1 marked fraction from wormwood, after addition of 1 µL of each fraction’s aqueous solution to the vessel, the end concentration (ng/mL) of each compound at 0.008 µg/mL of W1 consisted of 0.52 ± SD ng/mL chlorogenic acid, 0.03 ± SD ng/mL neochlorogenic acid, and 0.02 ± SD ng/mL 4-*O*-caffeoylquinic acid; meanwhile, at 0.8 µg/mL of W1, it consisted of 51.86 ± SD ng/mL chlorogenic acid, 2.99 ± SD ng/mL neochlorogenic acid, and 1.69 ± SD ng/mL 4-*O*-caffeoylquinic acid. The WS1 marked fraction from silver wormwood, at 0.008 µg/mL of WS1, consisted of 1.13 ± SD ng/mL chlorogenic acid, 0.05 ± SD ng/mL neochlorogenic acid, and 0.06 ± SD ng/mL 4-*O*-caffeoylquinic acid; meanwhile, at 0.8 µg/mL of WS1, it consisted of 112.52 ± SD ng/mL chlorogenic acid, 5.24 ± SD ng/mL neochlorogenic acid, and 6.11 ± SD ng/mL 4-*O*-caffeoylquinic acid.

**Figure A1:**
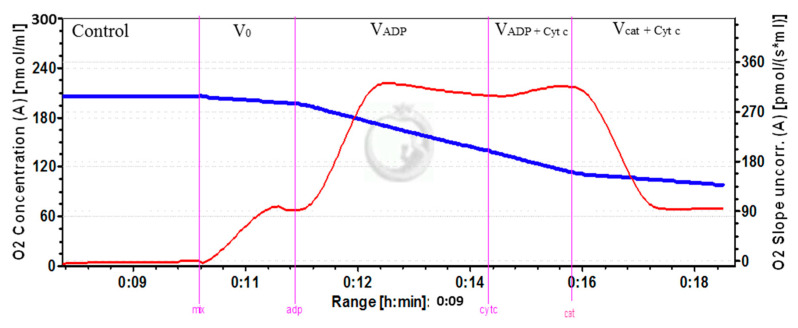
Original curve of kidney mitochondrial respiration in the control group.

In order to compare the direct effects of monocaffeoylquinic-acid-rich fractions from wormwood and silver wormwood herb acetone extracts (marked W1 and WS1, respectively) and dicaffeoylquinic fractions from the same (marked W2 and WS2, respectively) on mitochondrial oxidative phosphorylation, the mitochondria were incubated with two different concentrations (0.008 µg/mL and 0.8 µg/mL) of caffeoylquinic-acid-rich fractions and mitochondrial respiration rates in various metabolic states were measured. The original curve of kidney mitochondrial respiration in the control group (without caffeoylquinic-acid-rich fractions) is reported in Figure A1. As can be seen from Figure 3, all fractions of monocaffeoylquinic acids at both concentrations (0.008 µg/mL and 0.8 µg/mL) had no effect on routine (V_0_) (i.e., unrelated to ATP synthesis) respiration rate. Higher concentrations (0.8 µg/mL) of aqueous monocaffeoylquinic acid fractions reduced (W1—38%; WS1—15%) state 3 respiration rate (V_ADP_) as compared to the control group, *p* < 0.05 (Figure 3). 

**Figure 4:**
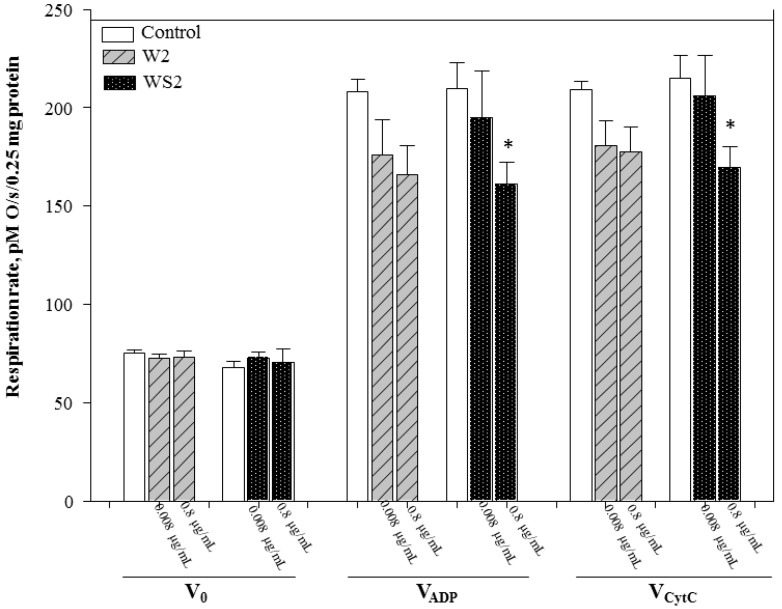
Effect of methanolic (dicaffeoylquinic-acid-rich) fractions from wormwood and silver wormwood herb acetone extracts on mitochondrial routine respiration rate, V_0_ (state 2 respiration rate in the presence of 0.25 mg/mL mitochondria and the substrates 5 mM glutamate + 5 mM malate), V_ADP_, (state 3 respiration rate in the presence of the substrates 5 mM glutamate + 5 mM malate and 1 mM ADP), and V_cyt C_ (state 3 respiration rate in the presence of 32 µM cytochrome c). *: *p* < 0.05 vs. respective control. In the W2 marked fraction from wormwood, after addition of 1 µL of each fraction’s aqueous solution to the vessel, the end concentration (ng/mL) of each compound at 0.008 µg/mL of W2 consisted of 0.14 ± SD ng/mL 4,5-dicaffeoylquinic acid, 1.00 ± SD ng/mL 3,4-dicaffeoylquinic acid, and 1.53 ± SD ng/mL 3,5-dicaffeoylquinic acid; meanwhile, at 0.8 µg/mL of W2, it consisted of 13.81 ± SD ng/mL 4,5-dicaffeoylquinic acid, 100.24 ± SD ng/mL 3,4-dicaffeoylquinic acid, and 152.69 ± SD ng/mL 3,5-dicaffeoylquinic acid. The WS2 marked fraction from silver wormwood, at 0.008 µg/mL of WS2, consisted of 0.82 ± SD ng/mL 4,5-dicaffeoylquinic acid, 1.43 ± SD ng/mL 3,4-dicaffeoylquinic acid, and 3.08 ± SD ng/mL 3,5-dicaffeoylquinic acid; meanwhile, at 0.8 µg/mL of WS2, it consisted of 81.70 ± SD ng/mL 4,5-dicaffeoylquinic acid, 143.02 ± SD ng/mL 3,4-dicaffeoylquinic acid, and 307.58 ± SD ng/mL 3,5-dicaffeoylquinic acid.

Dicaffeoylquinic-acid-rich fractions from wormwood and silver wormwood herb acetone extracts (marked W2 and WS2, respectively) also had no effect on routine respiration rate (V_0_). It is important to note that dicaffeoylquinic-acid-rich fractions (W2 and WS2) at both concentrations diminished state 3 respiration rate (V_ADP_) in a dose-dependent manner (Figure 4). There was no statistically significant effect of the dicaffeoylquinic-acid-rich fraction from wormwood herb acetone extract on state 3 respiration rate; the higher concentration of W2 reduced state 3 respiration rate (V_0_) less than the monocaffeoylquinic-acid-rich fraction from wormwood (marked W1). The higher concentration of the dicaffeoylquinic-acid-rich fraction from silver wormwood herb acetone extract (marked WS2) reduced state 3 respiration rate (V_ADP_) by 23%, *p* < 0.05 as compared to the control group (Figure 4). This concentration of fraction WS2 also reduced state 3 respiration rate more than the monocaffeoylquinic-acid-rich fraction from silver wormwood herb acetone extract (marked WS1).

**Figure 5:**
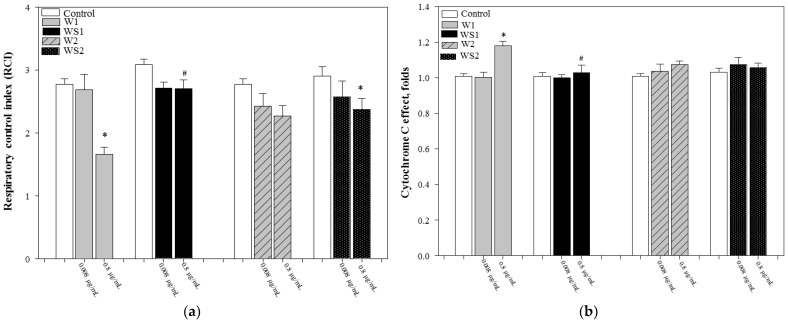
(**a**) Effect of fractions on the mitochondrial respiratory index with glutamate/malate as substrates. (**b**) Effect of fractions on the cytochrome c effect with glutamate/malate as substrates. * *p* < 0.05 vs. respective control; # *p* < 0.05 vs. fractions from wormwood (marked W1 and W2) at the same concentration. In the W1 marked fraction from wormwood, at 0.008 µg/mL of W1, the end concentration of compounds was 0.52 ± SD ng/mL chlorogenic acid, 0.03 ± SD ng/mL neochlorogenic acid, and 0.02 ± SD ng/mL 4-*O*-caffeoylquinic acid; meanwhile, at 0.8 µg/mL of W1, the end concentration of compounds was 51.86 ng/mL chlorogenic acid, 2.99 ± SD ng/mL neochlorogenic acid, and 1.69 ± SD ng/mL 4-*O*-caffeoylquinic acid. In the WS1 marked fraction from silver wormwood, at 0.008 µg/mL of WS1, the end concentration of compounds was 1.13 ± SD ng/mL chlorogenic acid, 0.05 ± SD ng/mL neochlorogenic acid, and 0.06 ± SD ng/mL 4-*O*-caffeoylquinic acid; meanwhile, at 0.8 µg/mL of WS1, the end concentration of compounds was 112.52 ± SD ng/mL chlorogenic acid, 5.24 ± SD ng/mL neochlorogenic acid, and 6.11 ± SD ng/mL 4-*O*-caffeoylquinic acid. In the W2 marked fraction from wormwood, at 0.008 µg/mL of W2, the end concentration of compounds was 0.14 ± SD ng/mL 4,5-dicaffeoylquinic acid, 1.00 ± SD ng/mL 3,4-dicaffeoylquinic acid, and 1.53 ± SD ng/mL 3,5-dicaffeoylquinic acid; meanwhile, at 0.8 µg/mL of W2, the end concentration of compounds was 13.81 ± SD ng/mL 4,5-dicaffeoylquinic acid, 100.24 ± SD ng/mL 3,4-dicaffeoylquinic acid, and 152.69 ± SD ng/mL 3,5-dicaffeoylquinic acid. In the WS2 marked fraction from silver wormwood, at 0.008 µg/mL of WS2, the end concentration of compounds was 0.82 ± SD ng/mL 4,5-dicaffeoylquinic acid, 1.43 ± SD ng/mL 3,4-dicaffeoylquinic acid, and 3.08 ± SD ng/mL 3,5-dicaffeoylquinic acid; meanwhile, at 0.8 µg/mL of WS2, the end concentration of compounds was 81.70 ± SD ng/mL 4,5-dicaffeoylquinic acid, 143.02 ± SD ng/mL 3,4-dicaffeoylquinic acid, and 307.58 ± SD ng/mL 3,5-dicaffeoylquinic acid.

The respiratory control indices decreased from 2.8 to 1.6 (W1, *p* < 0.05), 3.1 to 2.7 (WS1), 2.8 to 2.3 (W2), and 2.9 to 2.3 (WS2, *p* < 0.05) after the addition of two different concentrations of both caffeoylquinic-acid-rich fractions (Figure 5a). Furthermore, the cytochrome c effect obviously increased after the addition of higher concentrations of monocaffeoylquinic-acid-rich fractions (W1 and WS1). Higher concentrations of fraction W1 increased the cytochrome c effect by 1.20-fold compared to the control group; meanwhile, the higher concentration of fraction WS1 increased the cytochrome c effect by 1.03-fold compared to the control group, *p* < 0.05 (Figure 5b). During the experiments, an increasing tendency of the cytochrome c effect was also observed in the dicaffeoylquinic-acid-rich fraction groups; therefore, in further studies, we shall evaluate the cytochrome c reduction.

### 3.4. Measurement of the Cytochrome c Reduction Level of Caffeoylquinic-Acid Rich Fractions and Acetone Extracts

We determined the capacity of caffeoylquinic-acid-rich fractions to reduce cytochrome c. The absorbance spectrum of cytochrome c was measured in the range of 500–600 nm over time in the presence of two different concentrations (32.5 µg/mL and 65 µg/mL) of caffeoylquinic-acid-rich fractions.

**Figure 6:**
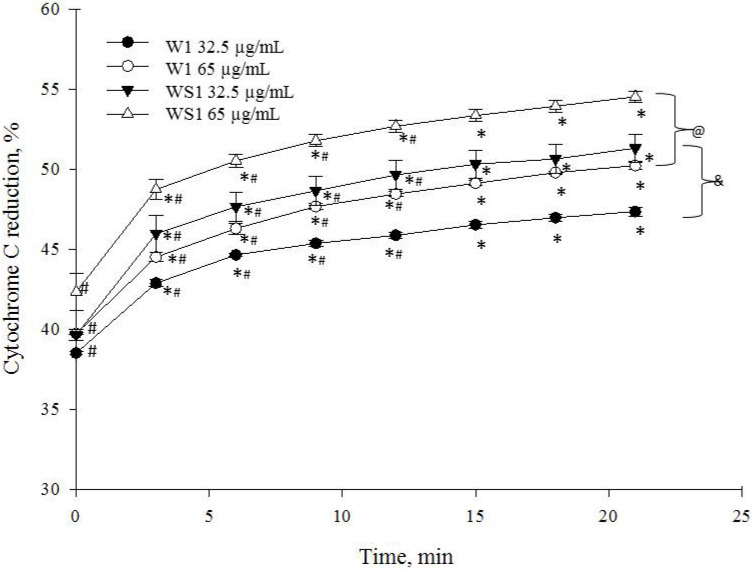
Effects of the monocaffeoylquinic-acid-rich fractions on cytochrome c reduction. * *p* < 0.05 at 0 min; ^#^ *p* < 0.05 at 21 min; ^@^ *p* < 0.05 vs. W1 at 32.5 µg/mL concentration; ^&^ *p* < 0.05 vs. WS1 at 65 µg/mL. In the W1 marked fraction from wormwood, at 32.5 µg/mL of W1, the end concentration of compounds was 2074.56 ± SD ng/mL chlorogenic acid, 119.53 ± SD ng/mL neochlorogenic acid, and 67.71 ± SD ng/mL 4-*O*-caffeoylquinic acid; meanwhile, at 65 µg/mL of W1, the end concentration of compounds was 4149.11 ± SD ng/mL chlorogenic acid, 239.05 ± SD ng/mL neochlorogenic acid, and 135.41 ± SD ng/mL 4-*O*-caffeoylquinic acid. In the WS1 marked fraction from silver wormwood, at 32.5 µg/mL of WS1, the end concentration of compounds was 4500.41 ± SD ng/mL chlorogenic acid, 209.71 ± SD ng/mL neochlorogenic acid, and 244.48 ± SD ng/mL 4-*O*-caffeoylquinic acid; meanwhile, at 65 µg/mL of WS1, the end concentration of compounds was 9000.81 ± SD ng/mL chlorogenic acid, 419.41 ± SD ng/mL neochlorogenic acid, and 488.96 ± SD ng/mL 4-*O*-caffeoylquinic acid.

We found that caffeoylquinic acids are potent cytochrome-c-reducing compounds. In the presence of 32.5 µg/mL and 65 µg/mL of the monocaffeoylquinic-acid-rich fraction from wormwood herb acetone extract (marked W1), there was fast and almost complete reduction of cytochrome c within 6–21 min of incubation; during the first minute it was 38.5 ± 0.1% and 39.8 ± 0.1%, respectively, reaching 44.7 ± 0.1% and 46.3 ± 0.4% after 6 min, respectively (Figure 6). The maximal reduction was achieved after 21 min (47.4 ± 0.3% and 50.2 ± 0.2%, respectively) (Figure 6), *p* < 0.05. The reducing capacity of 32.5 µg/mL and 65 µg/mL of the monocaffeoylquinic-acid-rich fraction from silver wormwood herb acetone extract (marked WS1) increased from 39.6 ± 0.7% and 42.4 ± 1.2%, respectively, during the first minute to 47.8 ± 1.4% and 50.5 ± 0.4%, respectively, after 6 min and reached the maximal reducing capacity (51.2 ± 1.1% and 54.5 ± 0.3%, respectively) at 21 min (Figure 6). 

**Figure 7:**
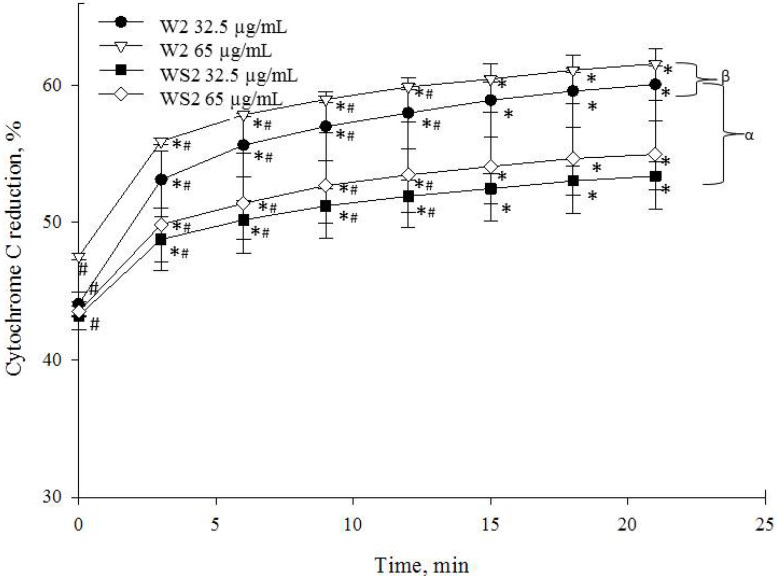
Effects of the dicaffeoylquinic-acid-rich fractions (W2 and WS2) on cytochrome c reduction. * *p* < 0.05 at 0 min; ^#^ *p* < 0.05 at 21 min; ^β^ *p* < 0.05 vs. WS2 at 32.5 µg/mL; ^α^ *p* < 0.05 vs. W2 at 65 µg/mL. In the W2 marked fraction from wormwood, at 32.5 µg/mL of W2, the end concentration of compounds was 552.42 ± SD ng/mL 4,5-dicaffeoylquinic acid, 4009.70 ± SD ng/mL 3,4-dicaffeoylquinic acid, and 6107.69 ± SD ng/mL 3,5-dicaffeoylquinic acid; Meanwhile, at 65 µg/mL of W2, the end concentration of compounds was 1104.83 ± SD ng/mL 4,5-dicaffeoylquinic acid, 8019.39 ± SD ng/mL 3,4-dicaffeoylquinic acid, and 12,215.38 ± SD ng/mL 3,5-dicaffeoylquinic acid. In the WS2 marked fraction from silver wormwood, at 32.5 µg/mL of WS2, the end concentration of compounds was 3268.09 ± SD ng/mL 4,5-dicaffeoylquinic acid, 5720.63 ± SD ng/mL 3,4-dicaffeoylquinic acid, and 12,303.19 ± SD ng/mL 3,5-dicaffeoylquinic acid; meanwhile, at 65 µg/mL of WS2, the end concentration of compounds was 6536 ± SD ng/mL 4,5-dicaffeoylquinic acid, 11,441.25 ± SD ng/mL 3,4-dicaffeoylquinic acid, and 24,606.37 ± SD ng/mL 3,5-dicaffeoylquinic acid.

The highest reducing capacity was observed in the dicaffeoylquinic-acid-rich fractions from wormwood and silver wormwood herb acetone extracts. The reducing capacity of 32.5 µg/mL of the dicaffeoylquinic-acid-rich fractions from wormwood and silver wormwood (marked W2 and WS2, respectively) increased from 53.1 ± 2.0% and 48.8 ± 1.7%, respectively, during the first minutes to 60.1 ± 3.0% and 53.4 ± 1.0% (at 21 min), respectively. Concentrations of 65 µg/mL of W2 and WS2 reduced cytochrome c by 55.9 ± 0.2% and 49.9 ± 3.3%, respectively, during the first minutes, and reached the maximal reducing capacity (61.6 ± 0.1% and 55.0 ± 4.0%, respectively) at 21 min (Figure 7). It should be noted that the reducing capacity of the dicaffeoylquinic-acid-rich fraction from silver wormwood herb acetone extract (marked WS2) was similar to that of the monocaffeoylquinic-acid-rich fraction from the same plant extract (marked WS1) (Figure 6 and Figure 7).

We also evaluated the cytochrome-c-reducing properties of wormwood and silver wormwood herb acetone extracts. The reducing capacity of 32.5 µg/mL herb acetone extracts from wormwood and silver wormwood (marked W and WS, respectively) increased from 40.3 ± 1.0% and 43.2 ± 0.4%, respectively, during the first minutes to 43.1 ± 1.4% and 47.3 ± 0.2% (at 21 min), respectively. Concentrations of 65 µg/mL of the W and WS extracts reduced cytochrome c by 41.2 ± 0.5% and 45.1 ± 0.4%, respectively, during the first minutes, and reached the maximal reducing capacity (44.5 ± 0.6% and 50.9 ± 0.4%, respectively) at 21 min (Figure S4). The reducing activity of herb acetone extract with both concentrations from wormwood was statistically significantly different from that of all caffeoylquinic-acid-rich fractions (*p* < 0.05).

## 4. Discussion

*Artemisia* species are important medicinal plants in various traditional medicine systems. As historical background is increasingly coupled with modern research, more scientific-based evidence is emerging that supports the pharmacological effects of diverse compounds present in the phytochemical profile of the species [6]. It is important to note that plants of the *Artemisia* genus are currently being extensively studied for their potential effects in treating COVID-19, in the form of the antimalarial drug artemisinin [29,30,31,32]. Recent data also show that catechol groups in caffeoylquinic acids may inhibit severe acute respiratory syndrome coronavirus 2 (SARS-CoV-2) [33]. Meanwhile, this study evaluated the antioxidant activity as well as the effect on kidney mitochondria and the cytochrome c reduction potential of the bioactive caffeoylquinic-acid-rich fractions extracted from cultivated wormwood and silver wormwood. Although numerous studies have shown a range of biological effects of caffeoylquinic acids obtained from different plant sources [34,35,36,37,38], there are no data regarding the comparison of mitochondrial-function-modulating properties of caffeoylquinic-acid-rich fractions obtained from wormwood and silver wormwood herb acetone extracts, despite the fact that interest in mitochondria-targeted natural antioxidants derived from plant materials has grown significantly in recent years. As far as we know, this is the first study on the effects of caffeoylquinic acid fractions purified from *Artemisia* herb acetone extracts on kidney mitochondrial functions and on cytochrome c redox state.

Phenolic profiles from the fractions obtained from wormwood and silver wormwood herb acetone extracts present caffeoylquinic acids as the predominant compounds. Fractionation resulted in mono- and dicaffeoylquinic-acid-specific extracts that displayed differences in mitochondria-targeted effects. The individual profiles of caffeoylquinic acids are species-dependent, and show significant individual quantitative and qualitative patterns [6]. Gouveia et al. performed quantification of caffeoylquinic acids from *Artemisia argentea*; they determined that the alcoholic extract from *A. argentea* consisted of various phenolic compounds—especially neochlorogenic acid (2.83 ± 0.02 mg/g), 3,5-dicaffeoylquinic acid (2.89 ± 0.01 mg/g) and 4,5-dicaffeoylquinic acid (0.28 ± 0.003 mg/g). Meanwhile, 3,4-dicaffeoylquinic acid was not detected [39]. Moeenfard et al. demonstrated that coffee brews can be considered as potential sources of antioxidants such as caffeoylquinic acids; they detected 44.51–818.93 mg/L chlorogenic acid, 16.21–409.02 mg/L neochlorogenic acid, and 12.57–444.69 mg/L 4-*O*-caffeoylquinic acid in various types of coffee brews [40]. In our study, we separated monocaffeoylquinic and dicaffeoylquinic acids from wormwood and silver wormwood herb acetone extracts, and determined larger amounts of them compared to the aforementioned studies. During fractionation, much larger amounts of caffeoylquinic acids were found in the aqueous and methanolic caffeoylquinic-acid-rich fractions compared to the wormwood and silver wormwood total herb acetone extracts. The aqueous monocaffeoylquinic-acid-rich fraction purified from wormwood (W1) herb acetone extract consisted of 66.8 ± 1.7 mg/g chlorogenic acid, 3.8 ± 0.1 mg/g neochlorogenic acid, and 2.2 ± 0.1 mg/g 4-*O*-caffeoylquinic acid, whereas the aqueous monocaffeoylquinic-acid-rich fraction purified from silver wormwood (WS1) herb acetone extract consisted of 143.3 ± 2.8 mg/g chlorogenic acid, 6.7 ± 0.1 mg/g neochlorogenic acid, and 7.8 ± 0.2 mg/g 4-*O*-caffeoylquinic acid. The methanolic dicaffeoylquinic-acid-rich fraction purified from wormwood (W2) herb acetone extract consisted of 122.3 ± 0.7 mg/g 3,4-dicafeoylquinic acid, 16.3 ± 0.6 mg/g 4,5-dicaffeoylquinic acid, and 186.5 ± 0.9 mg/g 3,5-dicaffeoylquinic acid, whereas the methanolic dicaffeoylquinic-acid-rich fraction purified from silver wormwood (WS2) herb acetone extract consisted of 175.9 ± 0.5 mg/g 3,4-dicaffeoylquinic, 101.1 ± 0.6 mg/g 4,5-dicaffeoylquinic acid, and 378.8 ± 0.5 mg/g 3,5-dicaffeoylquinic acid. These results show that the fractionation of caffeoylquinic acids by column chromatography is an appropriate method, resulting in the highest quantities of phenolic acids, as determined via the HPLC-PDA method. Furthermore, fractionation via column chromatography ensured the absence of other compounds (essential oil components) in the caffeoylquinic-acid-rich fractions from *Artemisia* herb acetone extracts.

Our study was focused on the fractionation of caffeoylquinic acids from *A. absinthium* and *A. ludoviciana* herb acetone extracts, and on the comparison of their antioxidant and mitochondria-targeted biological activity regarding the composition of monocaffeoylquinic and dicaffeoylquinic acids. We found that caffeoylquinic-acid-rich fractions from wormwood and silver wormwood herb extracts possess high antioxidant activity. The antioxidant activity of phenolic compounds depends on the arrangement of functional groups in the structure [41]. The caffeoyl group position and number on the quinic acid played a major role in the free radical scavenging activities of caffeoylquinic acids [33,42]. Phenolic acids—in our case, caffeoylquinic acids—display antioxidant activity as free radical scavengers or chelators, with exceptional effects on peroxynitrites and superoxide anions, as well as peroxyl and hydroxyl radicals [41]. Furthermore, caffeoylquinic acids have a greater ability to stabilize radicals due to the catechol group in their structures [43]. Olennikov et al. showed that *Artemisia frigida* herbal tea rich in caffeoylquinic and other phenolic acids is appropriate for use as a therapeutic and prophylactic measure for oxidative-stress-related pathologies. The two dominant phenolic acids of *A. frigida* herbal tea are 5-O-caffeoylquinic acid and 3,5-di-O-caffeoylquinic acid. According to DPPH analysis, the greatest radical scavenging potency was determined in five components of *A. frigida* herbal tea: 3,4,5-tricaffeoylquinic acid, 3,5-dicaffeoylquinic acid, 3,4-dicaffeoylquinic acid, 4,5-dicaffeoylquinic acid, and 5-O-caffeoylquinic acid [44]. We also found that the greatest ABTS radical scavenging potency was possessed by the dicaffeoylquinic-acid-rich fraction purified from silver wormwood herb acetone extract (marked WS2) (1693 ± 59 µM TE/g DW). Hong et al. state that the isolated 3,5-dicaffeoylquinic acid ethyl acetate fraction from methanolic leaf extract of *Ligularia fischeri* may be useful in developing effective anti-inflammatory medicaments. According to them, 3,5-dicaffeoylquinic acid showed strong DPPH, ABTS radical scavenging, and FRAP activity. 3,5-Dicaffeoylquinic acid effectively inhibited the production of NO and significantly repressed upregulation of inducible NO synthase, tumor necrosis factor-α, and cyclooxygenase-2 [45]. Li et al. also confirmed that dicaffeoylquinic acids possess radical scavenging and reducing capabilities via ABTS and FRAP assays [46]. In our study, the WS2 fraction, which contained the highest amount of 3,5-dicaffeoylquinic acid (378.8 mg/g DW), also showed strong ABTS radical scavenging and FRAP reducing activities. Hong et al. identified only 3,5-dicaffeoylquinic acid from the ethyl acetate fraction, while we identified 4,5-dicaffeoylquinic acid, 3,5-dicaffeoylquinic acid, and 3,4-dicaffeoylquinic acid from the 70% aqueous methanol fraction. Tajner-Czopek et al. studied the antioxidant activity of extracts derived from the medicinal plants coltsfoot, tarragon, lovage, white mulberry, caraway, and dandelion, described by their high quantities of caffeic acid derivatives. According to them, the highest antioxidant activity by ABTS assay was determined in the water-ethanol extract of tarragon, which contained the highest amount of 3,5-dicaffeoylquinic acid (18.58 mg caffeoylquinic acid × g^−1^)—a fast-acting antioxidant [47]—compared to other medicinal plants’ water or water-ethanol extracts [48]. These results from studies with caffeoylquinic-acid-rich fractions or extracts obtained from other plant sources are consistent with our results, which show that the greatest radical scavenging activity was observed in the methanolic dicaffeoylquinic-acid-rich fraction purified from silver wormwood herb acetone extract (marked WS2). On the other hand, according to Tamayose et al., quinic acid replaced at the C-4 position by a caffeoyl group showed a higher antiradical activity compared to C-5 or C-3 position acylation in monocaffeoylquinic acids [43]. In our research, a higher amount of 4-*O*-caffeoylquinic acid was found in the monocaffeoylquinic-acid-rich fraction purified from silver wormwood acetone extract (marked WS1) compared with the monocaffeoylquinic-acid-rich fraction from wormwood herb acetone extract (marked W1), while the radical scavenging activity of the former was also higher (745 ± 83 μM TE/g DW). Comparing dicaffeoylquinic acids with 3-caffeoylquinic acid (chlorogenic acid) and 5-caffeoylquinic acid (neochlorogenic acid), the dicaffeoylquinic acids possess higher antiradical activity due to the additional caffeoyl group in the structure esterified in the quinic acid, while those dicaffeoylquinic acids with the C-4 position of the caffeoyl group are more active [43]. Our results show that the dicaffeoylquinic-acid-rich fraction from silver wormwood herb acetone extract (marked WS2)*,* which was predominated by 4,5-dicaffeoylquinic acid and 3,4-dicaffeoylquinic acid, had the greatest antiradical activity (1693 ± 59 μM TE/g DW). The dicaffeoylquinic-acid-rich fraction purified from wormwood herb acetone extract (marked W2) had the lowest antiradical activity (679 ± 134 μM TE/g DW), which can be explained by the lower amounts of the aforementioned compounds. Tamayose et al. studied fractions from ethanolic extract of the aerial parts of *Moquiniastrum floribundum*, and determined that the ethyl acetate caffeoylquinic-acid-rich fraction possessed higher antiradical capacity [43]; these results are consistent with our study’s results.

It is well known that mitochondria not only produce ATP, but also generate ROS, and are involved in the regulation of apoptosis, regulation of intracellular Ca^2+^, and protection against infection [49]. Mitochondria are the most quantitatively important source of ROS—especially hydrogen peroxide (H_2_O_2_) and superoxide (O_2_**^●^**^−^) [50,51]; therefore, targeting mitochondria with antioxidants might be a promising strategy for potential therapeutic purposes in various pathologies. Because the caffeoylquinic-acid-rich fractions showed antioxidant activity, we evaluated their effects on mitochondrial function. Torres et al. indicated that the caffeoylquinic acids in acetone:water:lactic acid (40:60:1) extract from purple sweet potatoes’ tuberous roots have the potential to improve impaired mitochondrial function in hepatocytes and to increase fatty acid oxidation. They demonstrated that the addition of 5-caffeoylquinic acid or 3,4-dicaffeoylquinic acid increased the maximal respiration and the reserve respiratory capability in primary hepatocytes [52]. We found that caffeoylquinic-acid-rich fractions from wormwood and silver wormwood herb acetone extracts can modulate mitochondrial function, and that the effect on mitochondria is dose-dependent. Lower concentrations of both fractions purified from wormwood and silver wormwood herb acetone extracts had no effect on mitochondrial function, whereas higher concentrations (concentration of caffeoylquinic acids was at ng/mL range) caused inhibition of mitochondrial respiration rate at metabolic state 3. The aqueous monocaffeoylquinic-acid-rich fraction purified from wormwood herb acetone extract displayed higher suppressing activity (up to 38%). Accordingly, respiratory control index (RCI) was also found to decrease due to diminished State 3 respiration rate. Routine respiration rate (V_0_) was not changed, showing no changes in the permeability of the inner mitochondrial membrane. Moreover, we found that increasing the concentration of the monocaffeoylquinic-acid-rich fraction purified from wormwood herb acetone extract increased the effect of cytochrome c, i.e., increased the permeability of the outer mitochondrial membrane. Dicaffeoylquinic-acid-rich fractions purified from both wormwood and silver wormwood herb acetone extracts had no effect on the permeability of the outer mitochondrial membrane or on the release of cytochrome c. It is well known that cytochrome c is a component of the electron transport chain in the mitochondria that transports electrons from mitochondrial complex III to complex IV. As cytosolic cytochrome c might be rapidly reduced by various reductants or enzymes, regulation of the mitochondrial redox state of cytochrome c may modulate pathways of apoptosis [53,54]. Inhibiting apoptosis related to cytochrome c can block or slow the progression of various pathologies. To achieve this purpose, the biologically active compounds from plants should be applied in acute injury settings [55]. Therefore, we checked the ability of caffeoylquinic-acid-rich fractions purified from wormwood and silver wormwood herb acetone extracts to reduce cytochrome c even in the ng/mL concentration range. It is interesting to note that the caffeoylquinic acid fractions from both wormwood and silver wormwood herb contain potent cytochrome-c-reducing compounds, and effectively reduce cytochrome c. Dicaffeoylquinic-acid-rich fractions (4,5-dicaffeoylquinic acid, 3,4-dicaffeoylquinic acid, and 3,5-dicaffeoylquinic acid) exerted a greater cytochrome-c-reducing effect than that of monocaffeoylquinic-acid-rich fractions (chlorogenic acid, neochlorogenic acid, 4-*O*-caffeoylquinic acid) from wormwood and silver wormwood herb acetone extracts. The methanolic fraction from wormwood herb acetone extract (W2) had the greatest cytochrome-c-reducing effect (reduced cytochrome c by 55.9 ± 0.2% during the first minutes, and reached the maximal reducing capacity (61.6 ± 0.1%) at 21 min) compared with the methanolic fraction from silver wormwood herb acetone extract (WS2), *p* < 0.05. These results correlate with the FRAP assay results, which show that dicaffeoylquinic-acid-rich fractions purified from wormwood and silver wormwood herb acetone extracts have a strong ability to reduce Fe^3+^ into Fe^2+^. In the FRAP assay, the reducing activity of the methanolic fraction from wormwood (W2) herb acetone extract was the greatest of all (6952 ± 162 μM TE/g DW), *p* < 0.05. Moreover, our in vitro results using the FRAP method revealed that both fractions from both *Artemisia* species’ herb acetone extracts possess reducing properties. Therefore, we assume that *Artemisia* species fractions rich in caffeoylquinic acids can be suitable candidates for the modulation of mitochondrial function and regulation of mitochondrial redox state in the cells by suppressing ROS production and reducing cytochrome c. This finding is in agreement with our previous study showing that the ester of caffeic acid (caffeic acid phenethyl ester), which has a similar chemical structure to caffeoylquinic acids, also effectively reduces cytochrome c [28]. Our results are supported by the results of other scientists who have also investigated the role of cytochrome c redox state in the occurrence of apoptosis. Zazeri et al. indicated that quercetin possesses cytochrome-c-reducing properties, and that the potency of quercetin is related to its ability as a reducing agent and the appropriate molecular interplay between the protein and flavonoid [56]. The fact that dicaffeoylquinic-acid-rich fractions from *Artemisia* herb acetone extracts may regulate the mitochondrial redox state of cytochrome c is undoubtedly relevant for future research of mitochondrial-targeted plant compounds, because the regulation of the mitochondrial redox state of cytochrome c enables regulation of the intrinsic pathway of apoptosis, as was shown by Brown et al. in 2008 [54]. Thus, our present results may be relevant for the further investigation of caffeoylquinic-acid-rich fractions from *Artemisia* species herb extracts as a potential regulators of cytochrome c redox state and apoptosis. Banik et al. researched the effects of ethanolic *Trachyspermum ammi* L. (ajwain) extract on PC12 cells with cadmium-induced cytotoxicity; they indicated that treatment with Cd^2+^ at two different concentrations (5 and 10 μM) increased the relative cytochrome c levels in the cytosol of PC12 cells, though these higher levels of cytochrome c were decreased by the co-treatments of *T. ammi* extract. The release of cytochrome c into the cytosol from the mitochondria is a crucial stage in the apoptotic pathway of the mitochondria. According to Banik et al., *T. ammi* extract may potentially inhibit apoptosis [57]. Qian et al. also showed that aqueous extract of Danggui-Shaoyao-San—which consists of many plants, such as *Paeonia lactiflora*, *Ligusticum chuanxiong*, *Angelica sinensis*, *Poria cocos*, *Alisma orientale***,** and *Atractylodes macrocephala*—reduced the release of cytochrome c into the cytosol in PC12 cells treated with H_2_O_2_ [58].

**Figure 8:**
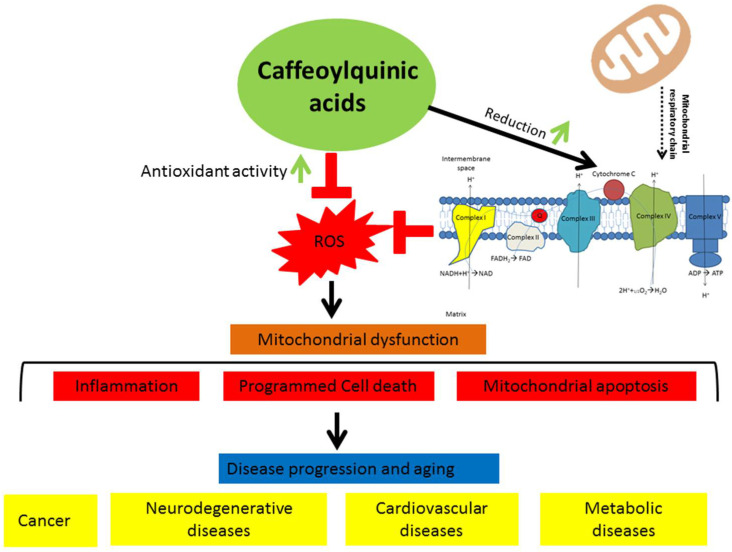
Possible mechanism of caffeoylquinic acids’ action in biological systems.

Our present study shows that aqueous and methanolic fractions purified from cultivated wormwood and silver wormwood herb acetone extracts are rich sources of monocaffeoylquinic and dicaffeoylquinic acids possessing high antioxidant (antiradical and reducing properties) activity, dose-dependent effects on mitochondrial functions, and cytochrome-c-reducing properties, but the further biological effects of these fractions and their pure compounds in vivo still remain to be examined. We especially think that the dicaffeoylquinic-acid-rich fractions purified from wormwood and silver wormwood herb acetone extracts could be a new source of natural biologically active components with antioxidant and cytochrome-c-reducing potential, and could be beneficial for regulating redox state in the cells, as well as in the modulation of the disturbed mitochondrial function in oxidative-stress-associated degenerative diseases, cancer, or cardiovascular and other diseases (Figure 8).

## 5. Conclusions

In the present study, we determined the profiles of the caffeoylquinic acids from purified fractions of cultivated wormwood (*A. absinthium*) and silver wormwood (*A. ludoviciana*) herb acetone extracts, along with their antioxidant activity (free radical scavenging activity; reducing activity), effects on mitochondrial function, and cytochrome-c-reducing properties.

The main findings of our study are as follows: (1) Aqueous fractions purified from wormwood and silver wormwood herb acetone extracts are rich in monocaffeoylquinic acids (chlorogenic acid, neochlorogenic acid, 4-*O*-caffeoylquinic acid), while methanolic fractions purified from wormwood and silver wormwood herb acetone extracts are rich in dicaffeoylquinic acids (4,5-dicaffeoylquinic acid, 3,4-dicaffeoylquinic acid, 3,5-dicaffeoylquinic acid). Aqueous fractions purified from wormwood and silver wormwood herb acetone extracts were solely composed of monocaffeoylquinic acids. Methanolic fractions purified from wormwood and silver wormwood herb acetone extracts contained only dicaffeoylquinic acids. (2) Both fractions purified from silver wormwood herb acetone extracts contained the greatest total content of caffeoylquinic acids. (3) The greatest radical scavenging activity was determined in the dicaffeoylquinic-acid-rich fraction purified from silver wormwood herb acetone extract; the greatest reducing activity was determined in the dicaffeoylquinic-acid-rich fraction purified from wormwood herb acetone extract. (4) The effect of fractions on mitochondrial function was dose-dependent; lower concentrations of caffeoylquinic-acid-rich fractions had no effect on mitochondrial function, whereas higher concentrations of caffeoylquinic-acid-rich fractions reduced state 3 respiration rate (with the complex-I-dependent substrate glutamate/malate). (5) Both monocaffeoylquinic- and dicaffeoylquinic-acid-rich fractions possess cytochrome-c-reducing properties; the highest cytochrome-c-reducing properties were observed in the dicaffeoylquinic-acid-rich fraction purified from wormwood herb acetone extract, which reduced cytochrome c by a greater extent. 

In summary, these findings show that caffeoylquinic acids might be beneficial as promising antioxidant agents for mitochondria-targeted medicine in the treatment of oxidative-stress-related degenerative illness and cancer. The results of our in vitro experiments revealed up-and-coming mitochondrial-function-modulating, antioxidant, and cytochrome-c-reducing properties of caffeoylquinic-acid-rich fractions from cultivated *Artemisia* species herb extracts based on several mechanisms of action. To better understand the mechanism of action and development of appropriate bioactive matters, further research and in vivo studies with animal models must be carried out.

## Figures and Tables

**Table 1:** The end concentration of each compound from purified caffeoylquinic-acid-rich fractions of wormwood (*A. absinthium*) and silver wormwood (*A. ludoviciana*) herb acetone extracts.

Compound	Concentration							
	0.008 µg/mL (ng/mL)				0.8 µg/mL (ng/mL)			
	Caffeoylquinic Acid-Rich Fractions							
	W1	WS1	W2	WS2	W1	WS1	W2	WS2
Chlorogenic acid	0.52 ± 0.024	1.13 ± 0.038	ND	ND	51.86 ± 2.362	112.52 ± 3.842	ND	ND
Neochlorogenic acid	0.03 ± 0.001	0.05 ± 0.002	ND	ND	2.99 ± 0.136	5.24 ± 0.179	ND	ND
4-*O*-caffeoylquinic acid	0.02 ± 0.001	0.06 ± 0.002	ND	ND	1.69 ± 0.077	6.11 ± 0.209	ND	ND
4,5-Dicaffeoylquinic acid	ND	ND	0.14 ± 0.009	0.82 ± 0.008	ND	ND	13.81 ± 0.898	81.70 ± 0.804
3,4-Dicaffeoylquinic acid	ND	ND	1.00 ± 0.010	1.43 ± 0.006	ND	ND	100.24 ± 0.967	143.02 ± 0.643
3,5-Dicaffeoylquinic acid	ND	ND	1.53 ± 0.012	3.08 ± 0.007	ND	ND	152.69 ± 1.177	307.58 ± 0.727

ND: not detected. Fraction W1 is the aqueous monocaffeoylquinic-acid-rich fraction from wormwood (*A. absinthium*) herb acetone extract; fraction WS1 is the aqueous monocaffeoylquinic-acid-rich fraction from silver wormwood (*A. ludoviciana*) herb acetone extract; fraction W2 is the methanolic dicaffeoylquinic-acid-rich fraction from wormwood (*A. absinthium*) herb acetone extract; fraction WS2 is the methanolic dicaffeoylquinic-acid-rich fraction from silver wormwood (*A.ludoviciana*) herb acetone extract.

**Table 2:** The end concentration of each compound from purified caffeoylquinic-acid-rich fractions from wormwood (*A. absinthium*) and silver wormwood (*A. ludoviciana*) herb acetone extracts.

Compound	Concentration							
	32.5 µg/mL (ng/mL)				65 µg/mL (ng/mL)			
	Caffeoylquinic Acid-Rich Fractions							
	W1	WS1	W2	WS2	W1	WS1	W2	WS2
Chlorogenic acid	2074.56 ± 95.95	4500.41 ± 156.07	ND	ND	4149.11 ± 191.90	9000.81 ± 312.14	ND	ND
Neochlorogenic acid	119.53 ± 5.53	209.71 ± 7.27	ND	ND	239.05 ± 11.06	419.41 ± 14.54	ND	ND
4-*O*-caffeoylquinic acid	67.71 ± 3.13	244.48 ± 8.48	ND	ND	135.41 ± 6.26	488.96 ± 16.96	ND	ND
4,5-Dicaffeoylquinic acid	ND	ND	552.42 ± 36.49	3268.09 ± 32.67	ND	ND	1104.83 ± 72.98	6536.18 ± 65.34
3,4-Dicaffeoylquinic acid	ND	ND	4009.70 ± 39.28	5720.63 ± 26.11	ND	ND	8019.39 ± 78.56	11,441.25 ± 52.22
3,5-Dicaffeoylquinic acid	ND	ND	6107.69 ± 47.82	12,303.19 ± 29.52	ND	ND	12,215.38 ± 95.65	24,606.37 ± 59.05

ND—not detected. Fraction W1 is the aqueous monocaffeoylquinic-acid-rich fraction from wormwood (*A. absinthium*) herb acetone extract; fraction WS1 is the aqueous monocaffeoylquinic-acid-rich fraction from silver wormwood (*A. ludoviciana*) herb acetone extract; fraction W2 is the methanolic dicaffeoylquinic-acid-rich fraction from wormwood (*A. absinthium*) herb acetone extract; fraction WS2 is the methanolic dicaffeoylquinic-acid-rich fraction from silver wormwood (*A. ludoviciana*) herb acetone extract.

**Table 3:** Antioxidant activity of fractions W1, WS1, W2, and WS2 from wormwood and silver wormwood herb acetone extracts.

Assay(μM TE/g DW)	W1	WS1	W2	WS2
ABTS	367 ± 58 ^#&@^	745 ± 83 *^@^	679 ± 134 *^@^	1693 ± 59 *^#&^
FRAP	5385 ± 168 ^&@^	5505 ± 175 ^&@^	6952 ± 162 *^#@^	6052 ± 81 *^#&^

* *p* < 0.05 vs. fraction W1; ^#^ *p* < 0.05 vs. fraction WS1; ^&^ *p* < 0.05 vs. fraction W2; ^@^ *p* < 0.05 vs. fraction WS2; fraction W1 is the aqueous monocaffeoylquinic-acid-rich fraction from wormwood herb acetone extract; fraction WS1 is the aqueous monocaffeoylquinic-acid-rich fraction from silver wormwood herb acetone extract; fraction W2 is the methanolic dicaffeoylquinic-acid-rich fraction from wormwood herb acetone extract; fraction WS2 is the methanolic dicaffeoylquinic-acid-rich fraction from silver wormwood herb acetone extract.

## Data Availability

All data is contained within the article and Supplementary Materials.

## Supplementary Materials

The following are available online at https://www.mdpi.com/article/10.3390/antiox10091405/s1: Figure S1: HPLC-PDA profiles at 320 nm, showing separation of phenolic acids in the total extract of wormwood herb; Figure S2: HPLC-PDA profiles at 320 nm, showing separation of phenolic acids in the total extract of silver wormwood herb; Figure S3: Example GC-MS profiles of caffeoylquinic-acid-rich fractions from wormwood and silver wormwood herb acetone extracts; Figure S4: Effects of acetone extracts from wormwood (W) and silver wormwood (WS) herb on cytochrome c reduction; Table S1: Total phenol content of total acetone extract, aqua, and methanol fractions from the herb of wormwood and silver wormwood, as measured by HPLC-PDA.

## Appendix A

**Table A1:** HPLC-PDA method quantification parameters, retention time, and UV-Vis absorption spectral data for identified compounds in total acetone extracts and fractions from wormwood and silver wormwood herb.

Compound	Calibration Curve	Coefficient of Determination (R^2^)	Rt (min)	λ max * (nm)
Chlorogenic acid	y = 47,700x + 2650	0.99992	14.41	245 sh, 296 sh, **325**
Neochlorogenic acid	y = 25,600x + 1730	0.99944	10.52	245 sh, 296 sh, **325**
4-*O*-caffeoylquinic acid	y = 60,200x − 1620	0.99966	14.95	245 sh, 296 sh, **325**
4,5-Dicaffeoylquinic acid	y = 40,400x − 9600	0.99997	30.98	245 sh, 296 sh, **325**
3,4-Dicaffeoylquinic acid	Y = 56,700x − 6790	0.99999	35.35	245 sh, 296 sh, **327**
3,5-Dicaffeoylquinic acid	y = 79,100x − 3690	0.99989	34.02	245 sh, 296 sh, **327**
Luteolin-7-glucoside	y = 54,300x + 1040	0.99998	26.92	254 sh, 267 sh, **348**
Luteolin-7-rutinoside	y = 40,300x − 3980	0.99999	23.80	254 sh, 267 sh, **348**

* The wavelengths of maximum absorption are in bold; sh: shoulder.
