# Supplementary material for: Antioxidant and Mitochondria-Targeted Activity of Caffeoylquinic-Acid-Rich Fractions of Wormwood (Artemisia absinthium L.) and Silver Wormwood (Artemisia ludoviciana Nutt.)
Source: Antioxidants (Basel). 2021 Sep 1;10(9):1405. doi: 10.3390/antiox10091405 (PMC8469600; doi:10.3390/antiox10091405)
Supplement: Supplementary file 1 [file antioxidants-10-01405-s001.zip › antioxidants-1335377-supplementary.pdf]

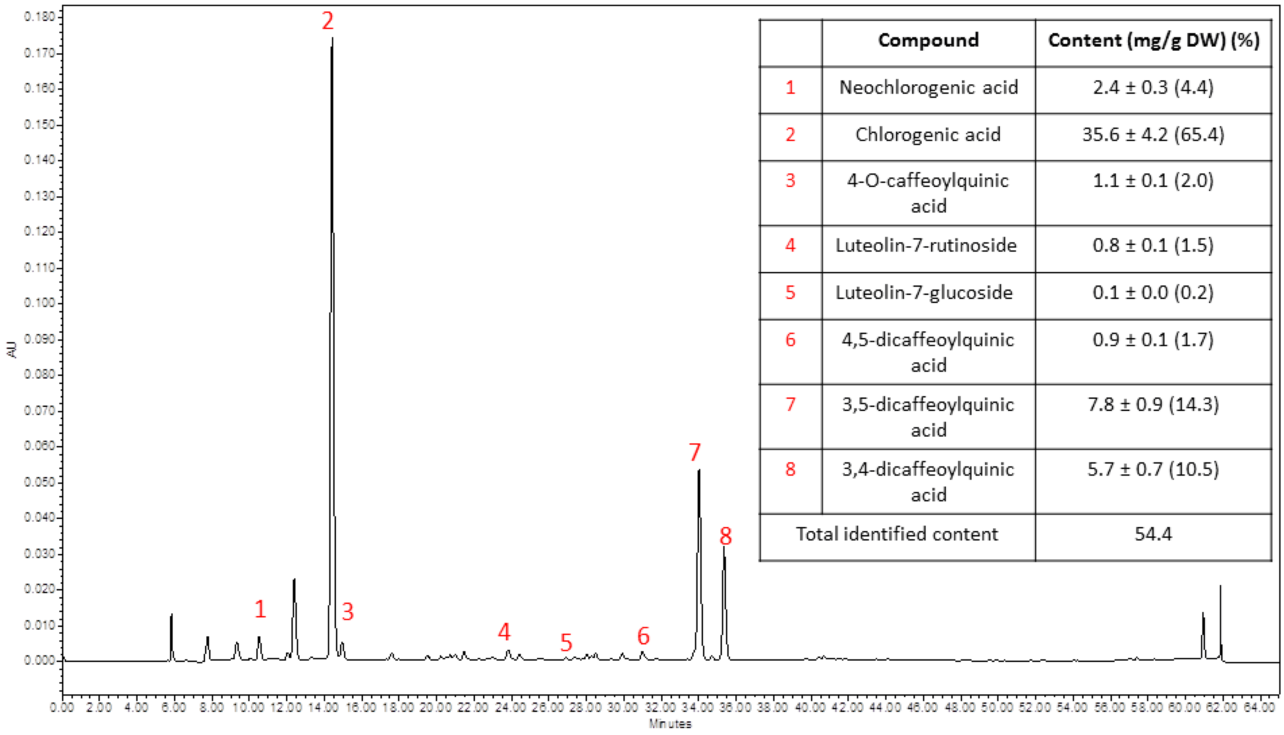

	Compound	Content (mg/g DW) (%)
1	Neochlorogenic acid	2.4 ± 0.3 (4.4)
2	Chlorogenic acid	35.6 ± 4.2 (65.4)
3	4-O-caffeoylquinic acid	1.1 ± 0.1 (2.0)
4	Luteolin-7-rutinoside	0.8 ± 0.1 (1.5)
5	Luteolin-7-glucoside	0.1 ± 0.0 (0.2)
6	4,5-dicaffeoylquinic acid	0.9 ± 0.1 (1.7)
7	3,5-dicaffeoylquinic acid	7.8 ± 0.9 (14.3)
8	3,4-dicaffeoylquinic acid	5.7 ± 0.7 (10.5)
Total identified content		54.4

Figure S1. HPLC-PDA profiles at 320 nm, showing phenolics separation in the total acetone extract of wormwood herb.

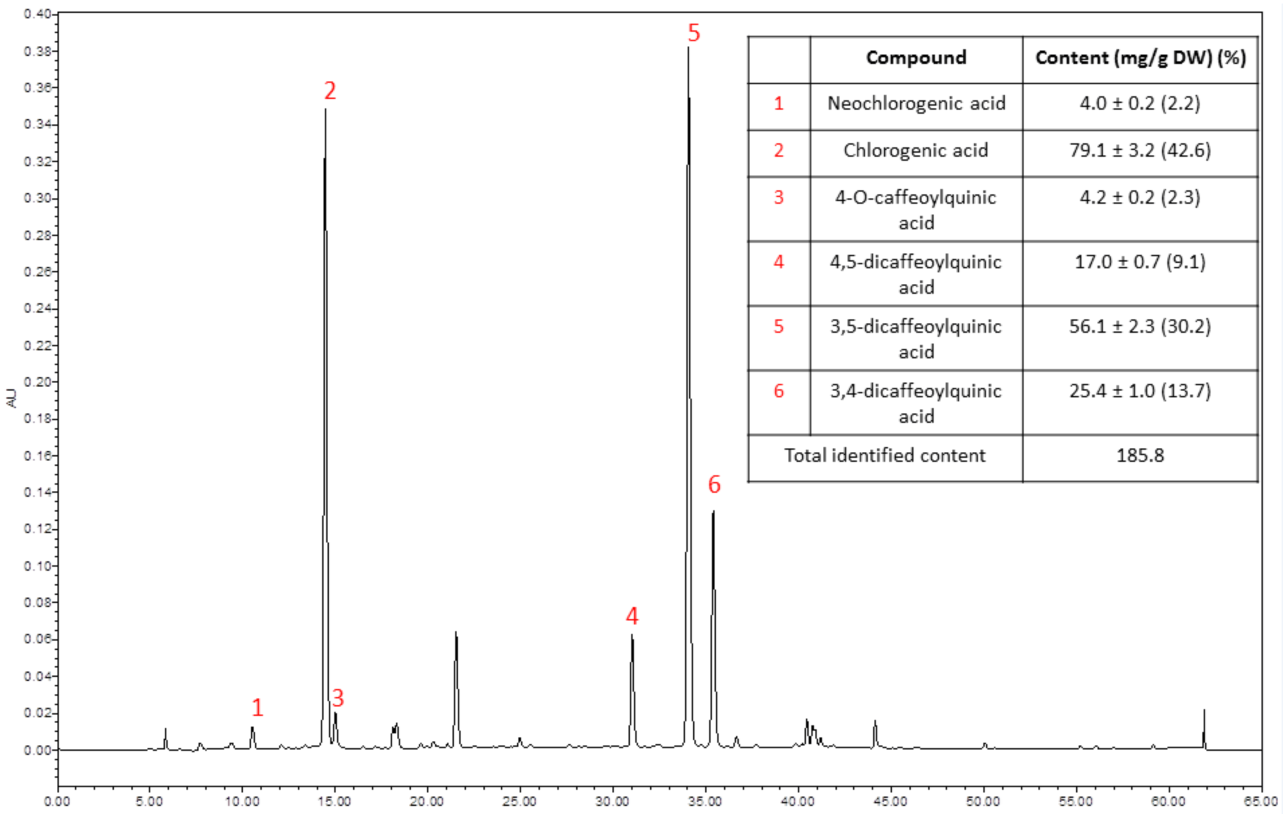

	Compound	Content (mg/g DW) (%)
1	Neochlorogenic acid	4.0 ± 0.2 (2.2)
2	Chlorogenic acid	79.1 ± 3.2 (42.6)
3	4-O-caffeoylquinic acid	4.2 ± 0.2 (2.3)
4	4,5-dicaffeoylquinic acid	17.0 ± 0.7 (9.1)
5	3,5-dicaffeoylquinic acid	56.1 ± 2.3 (30.2)
6	3,4-dicaffeoylquinic acid	25.4 ± 1.0 (13.7)
Total identified content		185.8

Figure S2. HPLC-PDA profiles at 320 nm, showing phenolics separation in the total acetone extract of silver wormwood herb.

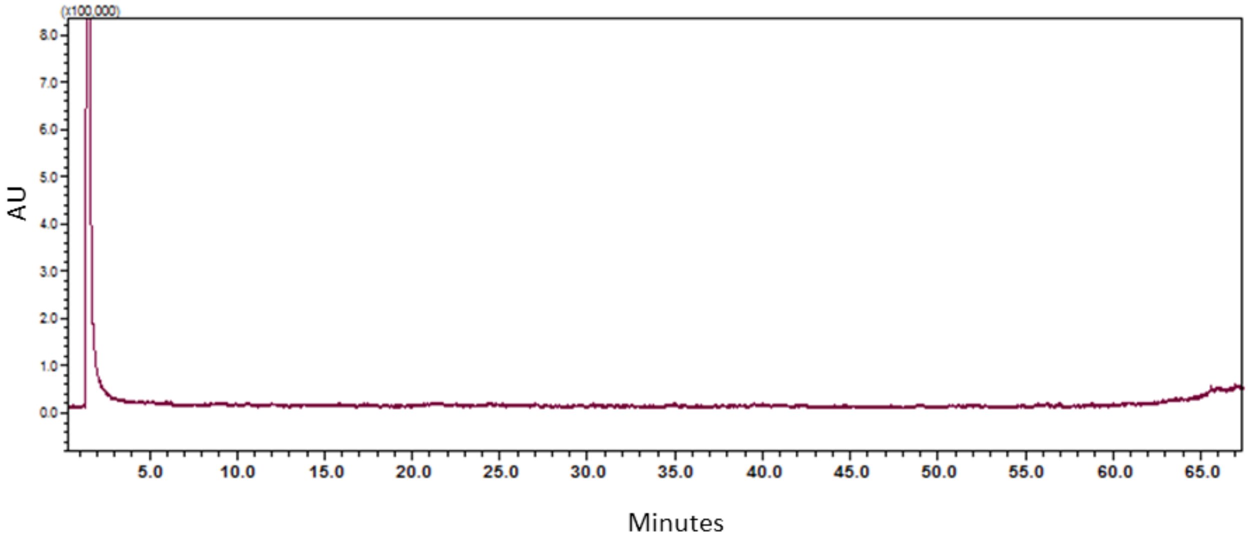

Figure S3. Example GC-MS profile of caffeoylquinic acid-rich fractions from wormwood and silver wormwood herb acetone extracts.

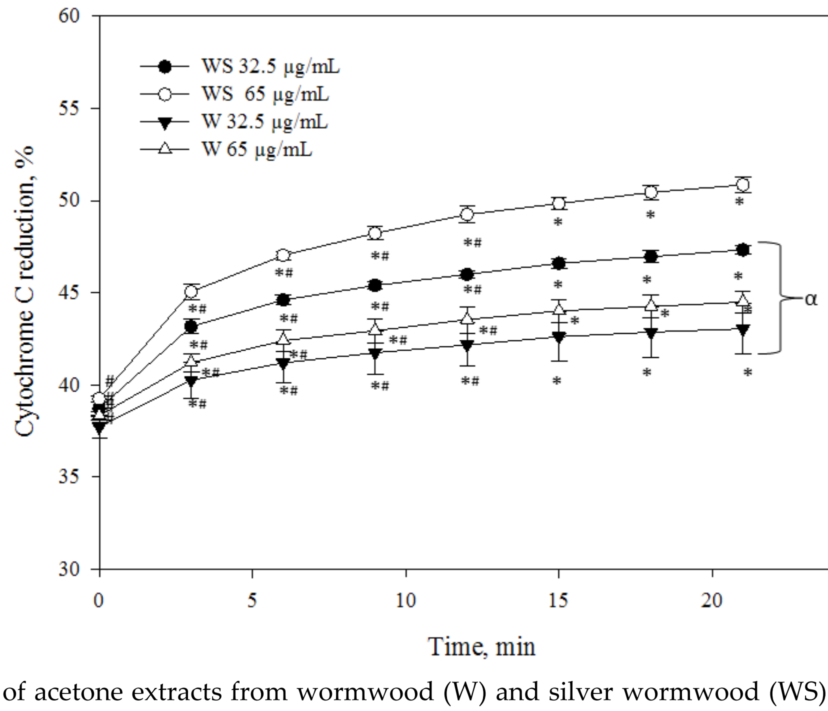

Figure S4. Effect of acetone extracts from wormwood (W) and silver wormwood (WS) herb on cytochrome c reduction. * $p < 0.05$ vs. at 0 min time; $^{\#}$ $p < 0.05$ vs. at 21 min time; $^{\alpha}$ $p < 0.05$ vs. 65 µg/mL acetone extract from silver wormwood herb. WS marked acetone extract from silver wormwood herb, at 32.5 µg/mL of WS the end concentration of compounds was consisted of 2572.32 ± 104.08 ng/mL chlorogenic acid, 131.62 ± 5.33 ng/mL neochlorogenic acid, 136.80 ± 5.54 ng/mL 4-O-caffeoylquinic acid, 552.87 ± 22.37 ng/mL 4,5-dicaffeoylquinic acid, 825.28 ± 33.39 ng/mL 3,4-dicaffeoylquinic acid, 1823.48 ± 73.78 ng/mL 3,5-dicaffeoylquinic acid. At 65 µg/mL of WS the end concentration of compounds was consisted of 5144.64 ± 208.17 ng/mL chlorogenic acid, 263.25 ± 10.65 ng/mL neochlorogenic acid, 273.60 ± 11.07 4-O-caffeoylquinic acid, 1105.74 ± 44.74 ng/mL 4,5-dicaffeoylquinic acid, 1650.56 ± 66.79 ng/mL 3,4-dicaffeoylquinic acid, 3646.97 ± 147.57 ng/mL 3,5-dicaffeoylquinic acid. W marked acetone extract from wormwood herb, at 32.5 µg/mL of W the end concentration of compounds was consisted of 1155.71 ± 136.39 ng/mL chlorogenic acid, 77.57 ± 9.16 ng/mL neochlorogenic acid, 34.58 ± 4.08 ng/mL 4-O-caffeoylquinic acid, 30.44 ± 3.59 ng/mL 4,5-dicaffeoylquinic acid, 184.76 ± 21.80 ng/mL 3,4-dicaffeoylquinic acid,

254.03 ± 29.98 ng/mL 3,5-dicaffeoylquinic acid, 4.25 ± 0.50 ng/mL luteolin-7-glucoside, 38.83 ± 4.58 ng/mL luteolin-7-rutenoside. At 65 μg/mL of W the end concentration of compounds was consisted of 2311.42 ± 272.79 ng/mL chlorogenic acid, 155.15 ± 18.31 ng/mL neochlorogenic acid, 69.16 ± 8.16 ng/mL 4-O-caffeoylquinic acid, 60.88 ± 7.19 ng/mL 4,5-dicaffeoylquinic acid, 369.52 ± 43.61 ng/mL 3,4-dicaffeoylquinic acid, 508.06 ± 59.96 ng/mL 3,5-dicaffeoylquinic acid, 8.50 ± 1.00 ng/mL luteolin-7-glucoside, 77.66 ± 9.17 ng/mL luteolin-7-rutinoside.

Table S1. Total phenol content of total acetone extract, aqua and methanol fractions from the herb of wormwood and silver wormwood by HPLC-PDA method.

Extract	Total identified phenolic mg/g DW	
	**Wormwood**	**Silver wormwood**
**Acetone (total)**	54.4	185.8
**Aqua**	72.8	157.8
**Methanol**	325.1	655.8
